# Northern Indiana and Southern Michigan Society

**Published:** 1895-11

**Authors:** 


### NORTHERN INDIANA AND SOUTHERN MICHIGAN SOCIETY.

The ninth semi-annual session of the Northern Indiana and Southern Michigan Homœopathic Medical Association was held October 15th, in Goshen, Ind., with Dr. T. C. Buskirk, in the absence of the President, Dr. I. O. Buchtel, Auburn, in the chair. Members present: Drs. E. W. Sawyer, Chicago; T. C. Buskirk, White Pigeon; W. H. Shaw, Constantine; Geo. A. Whippy, Ligonier; John Borough, Mishawaka; J. E. Barbour, Bristol; A. L. Fisher, Porter Turner, and H. A. Mumaw, Elkhart; W. A. Whippy and W. B. and M. K. Kreider, Goshen. Dr. C. F. Ellis, Eureka Springs, Ark., was present as a visitor, but took active part in the discussions.

A number of papers from absent members and friends of the Association were read, expressing regrets at their inability to be present. The meeting was called to order at 1 P. M. by the second Vice-President, and after roll call the minutes of the previous meeting were read by the Secretary, Dr. H. A. Mumaw, and approved. After the report of the Treasurer, necrologists, censors, delegates from other societies, and collection of annual dues, the regular work of the Association was in order. First, reports of bureaux: Surgery, Dr. Turner; Ophthalmology, Dr. W. B. Kreider; Materia Medica, Dr. Franz; Practice, Dr.

Shaw; Gynecology, Dr. M. K. Kreider; Pædology, Dr. Leib. The following papers were read and fully discussed by all the members present: "Laryngeal Diphtheria," Dr. Turner; "Middle Ear Diseases Resulting from La Grippe," Dr. W. B. Kreider; "Two Cases of Malignant Diseases of the Uterus," Dr. Fisher; "Vaccination—Dangerous Grounds," Dr. Shaw; "Gynecological Cases," Dr. M. K. Kreider; "Constipation of Infants," Dr. Buskirk. Reports of cases—Drs. Ellis, Sawyer, Barbour, Mumaw, and Kreider. Chairmen of bureaus for the next meeting were appointed as follows: Surgery, Dr. Buchtel; Ophthalmology, Dr. W. B. Kreider; Materia Medica, Dr. Geo. Whippy; Practice, Dr. Barbour; Gynecology, Dr. Franz; Pædology, Dr. Turner.

The meeting was one of special interest and profit. It was unanimously decided to hold the next meeting at Elkhart on the first Tuesday in May, 1896. The meeting then adjourned.

